# Supplementary material for: Views of medical residents on a research training program: A qualitative study
Source: PLoS One. 2022 Jan 21;17(1):e0261583. doi: 10.1371/journal.pone.0261583 (PMC8782500; doi:10.1371/journal.pone.0261583)
Supplement: S1 Table — (DOCX) [file pone.0261583.s001.docx]

**Tables**

**Table 1:** Participant comments pertaining to expectations of residents, program coordination, mentorship, and capacity building

| Participants | Comments pertaining to ***expectations of residents*** |
| --- | --- |
| FG1-P01 | ‘*some residents had more experience than others’* |
| FG1-P02 | ‘residents lacked *ABCs of research’* |
| FG1-P03 | *“I had my own idea and I worked on the literature review from scratch. When I proposed the topic to my attending, he told me this wouldn’t be a high impact study”* |
| FG1-P01 | *“We were doing the data sheet and h­­­e asked me to do the table and put the values for the research, I wasn’t sure, should I put them in a horizontal or vertical way, divide them by sex or just put them all together, I really did not know”* |

| Participants | Comments pertaining to ***program coordination*** |
| --- | --- |
| FG1-P04 | “resident identifies the faculty member *as a navigator*” |
| FG1-P05 | *“I checked my specialty, and the people who are active in this research and I sent emails.”* |
| FG1-P06 | *“Some programs might be three, four or five years but overall enough to choose and initiate a study and to change, trial and error, if you don’t like your study you can change because you have a lot of time*” |
| FG1-P07 | *“Time limits that you have either for 6 or 12 months for your abstract or your proposal in one year should be done. There’s a timeline that you must follow. I think that’s very good for us specially that doctors get overwhelmed with the time. Follow up is also one of the main advantages that we have”* |
| FG1-P04 | *“portal to upload our proposal... it’s a good way”* |
| FG1-P08 | “W*e don’t need 2 years to present the proposal, it’s a bit too much. We were told about the timeline when we started residency, but we just lose track.”* |
| FG2-P09 | *“It was very long in the beginning and then at the last year the deadlines become very intense. Condensed… And then we got stuck during the last year*” |
| FG1-P01 | *“If something went wrong during data collection, in the third year, I have to go back from the beginning, so how am I going to make any progress in a new project if I’m going to start in mid third year and I’m not allowed to graduate if I don’t have a research project*” |
| FG2-P09 | *“Make the deadlines sooner, for example for the proposal, it was 2 years to get the proposal, it’s a bit too much* |
| FG1-P04 | *“Shift everything 6 months”* |
| FG2-P09 | *“Shift everything back. Make the deadlines closer”* |
| FG1-P01 | *“2 person/project structure: less burden than that experienced by one person. You would decrease the workload, and at the same time you would have your name in publication”* |
| FG1-P04 | “*a reference person*” |
| FG1-P08 | *“In every unit we have a chairman so why don’t we have a person who is the head of research in the department and asks people where you are now”* |
| FG2-P10 | *“And you would rather that person be an attending?”* |
| FG1-P08 | *“Yes because, “maha” only follows the residents. maybe this champion meets with the residents and attending at the same time and ask them where are you at.”* |
| FG1-P02 | *“A closer follow up. Where are we right now what did we do so far, a close face to face interaction, a regular follow up”* |

| Participants | Comments pertaining to ***mentorship*** |
| --- | --- |
| FG1-P05 | *“Most of them didn’t reply. I went to the one who replied and talked to him, he was very cooperative”* |
| FG2-P11 | *“I asked my seniors whom they worked with and who was most dedicated and engaged in research and who is most cooperative to work with and who finds time most to work”* |
| FG1-P04 | *“If someone asked me what you think about choosing this mentor over someone else, if he has a research assistant I will say go for it.”* |
| FG1-P04 | *“Again as P07 said we are always more than encouraged to choose our own topic rather than going to the mentor and asking them what they have as a potential project”* |
| FG2-P12 | *“FRRP program tells you that you can choose whoever you want but when you get to actually doing it you are limited by the specialty and sometimes one person within that specialty”* |
| FG1-P02 | *“Sometimes we really try to be on track, we always send them that we need to finish but due to his busy schedule or when he’s abroad for a conference, he keeps on postponing the issue. It is important to stress that there are deadlines to be part of the whole procedure as well”* |
| FG2-P09 | *“Took more than 6 to 7 months to get it back.”* |
| FG1-P03 | *“Mentors are not available or already saturated with their mentees, so we find ourselves limited.”* |
| FG1-P03 | “*worrying about disappointing the mentors*” |
| FG2-P13 | “*acting like you know how you are doing*” |
| FG1-P03 | *“Sometimes I would go on the internet, on google and YouTube”* |
| FG2-P13 | *“An attending asks have you done it before? if you say no, he won’t give it to you. If you’ve read it maybe and seen videos a million times but you show confidence in front of him, he would trust you”* |
| FG1-P04 | Most feel embarrassed to ask “*silly questions*” |
| FG1-P01 | avoid being judged as not competent enough for the program so they “*rather not ask*” |
| FG1-P01 | *“My mentor asked me where you are now with the research, I said it’s in progress and that I’m trying to get the IRB and that was it. He writes FRRP in progress, and honestly at some point, you might feel shy to ask the attending about something.”* |
| FG1-P06 | *“They [mentors] know more than us, they have their experiences and can help us.”* |
| FG1-P07 | *“The idea of having a topic by ourselves shouldn’t be the focus for FRRP it’s more like to learn how to start a research from scratch. Regardless of the topic by itself”* |
| FG1-P07 | *“The list of advisors, do not include all people who are dedicated for research, because we know in every specialty the attending who is very busy doing always research”* |
| FG2-P12 | *“You are asking FRRP to indicate who are the good attendings.”* |
| FG2-P12 | *“Ensure that the mentor is available to the mentee and you’re proposing one way is to stick the number of mentees to that person”* |

| Participants | Comments pertaining to ***capacity building*** |
| --- | --- |
| FG1-P06 | *“The topics chosen are very good; manuscript writing, data analysis, SPSS, proposal, and I think they are very good. Instead of increasing the series of lectures, maybe that time will be allocated for research. I don’t think adding on the very good topics will add any benefit or time efficiency to the residents.”* |
| FG1-P02 | *“Certain things may actually rise; several people would actually agree and it also happened with me”* |
| FG1-P08 | *“When it’s a small group it’s easier to understand and we can teach other it would be fine.”* |
| FG2-P14 | *“We have sessions on moodle, that you can access. I think they are useful as a reminder after attending the lecture and we’re having any question we can use it.”* |
| FG2-P11 | *“It’s good to be exposed to this kind of work because many residents are speakers abroad, they prepare posters, and go to national conferences and meetings so it’s like an exercise.”* |
| FG1-P08 | *“The open house actually mimics the conferences”* |
| FG2-P11 | *“yeah”* |
| FG1-P08 | *“It’s a good opportunity for you then to be part of it and experience that whole poster presentation and oral presentation”* |
| FG2-P11 | *“Exactly”* |
| FG2-P11 | *“We set meetings whenever we have issues. They always find time available. Whenever the attending is there, we can ask questions, we just refer to them, what should I do about this, and they directly give us an answer. Last time I had a question for [mentor], she picked up the phone and called [FRRP person]. Another time someone else and she solved the issue, it was very easy.”* |
| FG1-P04 | *The sessions: “But it’s not interactive”* |
| FG1-P04 | *“But honestly we don’t check them”* |
| FG2-P11 | *“The only time we have dedicated to education is our morning lecture, from 7 till 7:30 am this is the only time, I’m sure we can make it at another time otherwise, I don’t know”* |
| FG1-P01 | *“I think there has to be something at the beginning to introduce research”* |
| FG1-P04 | “*a standard like first 6 months to a year where all the specialties are to attend 2 or 3 workshops*” |
| FG1-P04 | “*even if you are not going to do research later on, the FRRP helps you understand how research is conducted and how it should be interpreted*” |
| FG2-P13 | *“We have to generalize the ABCs, like we said grand rounds for everyone, and then after a certain point this has to become personalized, individualized, so that there is this one reference person that whenever you have any question you can just go and they are ready to help”* |
| FG2-P13 | “*I think that FRRP is not something that would help me it’s more of something forcing me to do a research before I graduate… it’s just something I have to do because it is mandatory to do it*” |
| FG1-P01 | “*optional*” |
