# Supplementary material for: Views of medical residents on a research training program: A qualitative study
Source: PLoS One. 2022 Jan 21;17(1):e0261583. doi: 10.1371/journal.pone.0261583 (PMC8782500; doi:10.1371/journal.pone.0261583)
Supplement: S1 Appendix — (DOCX) [file pone.0261583.s002.docx]

**Appendices**

Appendix 1: interview guide

The start:

Each resident introduces himself, states his area of interest and previous research experience

1. What are your thoughts about the FRRP experience?

Probing:

Do you think that the FRRP provided you with an adequate research experience?

How do you perceive your skills and knowledge in the research steps (literature review, defining a research question, writing a proposal, collecting data, analyzing data, writing a report, delivering oral PPT, preparing a poster) *pre- and post- FRRP*?

How do you define a mentor?

Do you think your mentor had enough time to allocate for you and to guide you through the process?

Do you think that the FRRP gave you the opportunity to develop a long-term professional relationship with your advisor?

1. How do you perceive a perfect FRRP program?

Probing:

Do you think residents should be given an allocated time to perform research?

Did you take the course on the research process posted on moodle for FRRP residents?

Do you think additional sessions are needed?

Do you prefer hands-on or recoded sessions?

In your opinion, in which steps of the research process residents need help? How can we offer help?

What specific aspects of the program could be improved?

In your opinion, how can we increase FRRP publications?
