# Supplementary material for: Views of medical residents on a research training program: A qualitative study
Source: PLoS One. 2022 Jan 21;17(1):e0261583. doi: 10.1371/journal.pone.0261583 (PMC8782500; doi:10.1371/journal.pone.0261583)
Supplement: Attachment — Submitted filename: Response to Reviewers.docx [file pone.0261583.s003.docx]

**Department of Internal Medicine**

**American University of Beirut**

December 2, 2021

Re: Views of medical residents on a research training program: a qualitative study

Dear Dr. Emily Chenette,

Thank you for the opportunity to revise and resubmit our above titled manuscript to PLOS ONE. We thank you and the reviewers for taking the time to review the manuscript. We found the comments and suggestions very constructive and used them to improve our manuscript. We are hopeful this study will be useful as research programs in different hospitals are launched.

Kindly find below a point-by-point response to the reviewers’ comments and a description of the changes made to the manuscript.

Competing interest statement: Dr. Elie Akl directs the Clinical Research Institute, under which the program that was evaluated operates. Dr. Hani Tamim directs the Fellowship and Residency Research Program, the program that was evaluated. Ms. Abi Khalil coordinates the Fellowship and Residency Research Program, the program that was evaluated. All remaining authors have nothing to disclose. Please note that this does not alter our adherence to PLOS ONE policies on sharing data and materials.

We look forward to the outcome of the peer review of our revised manuscript.

Sincerely,

Elie A. Akl, MD, MPH, PhD

AUBMC, Department of Internal Medicine

P.O. Box: 11-0236

Riad-El-Solh Beirut 1107 2020

Beirut – Lebanon

Phone: 00961 1 350 000

[ea32@aub.edu.lb](mailto:ea32@aub.edu.lb)

# Response to Reviewers

The reviewers’ comments are listed below in bold. Our responses to these comments are in regular font. We have indicated the sections where revisions have been made in our manuscript.

1. **Please ensure that your manuscript meets PLOS ONE's style requirements, including those for file naming.**

**Response:** We have revised the manuscript to ensure it meets PLOS ONE's style requirements

2. **Thank you for stating the following in the Competing Interests section:
   [-Dr. Elie Akl directs the Clinical Research Institute, under which the program that was evaluated operates.
   -Dr. Hani Tamim directs the Fellowship and Residency Research Program, the program that was evaluated.
   -Ms. Abi Khalil coordinates the Fellowship and Residency Research Program, the program that was evaluated.
   -All remaining authors have nothing to disclose.]**

**Please confirm that this does not alter your adherence to all PLOS ONE policies on sharing data and materials, by including the following statement: "This does not alter our adherence to  PLOS ONE policies on sharing data and materials.” (as detailed online in our guide for authors http://journals.plos.org/plosone/s/competing-interests).  If there are restrictions on sharing of data and/or materials, please state these. Please note that we cannot proceed with consideration of your article until this information has been declared.**

**Response:** Thank you. We have included the above statement in the manuscript following the Competing Interests section.

**Please include your updated Competing Interests statement in your cover letter; we will change the online submission form on your behalf.**

**Response:** We have included the updated Competing Interests statement in our cover letter.

3. **Please include your tables as part of your main manuscript and remove the individual files. Please note that supplementary tables (should remain/ be uploaded) as separate "supporting information" files**

**Response:** The one table we had included in the first version was a very large one that we opted to include as a “supporting information” file. We have uploaded it separately. We might have created confusion by previously naming it table 1. We have now included another table in the main manuscript (please see below).

**Reviewer #1**

**The methodology of this article is sound however the intent and usefulness of this articles for other educators is not clear.** **This article would be significantly strengthened if it was written with outward intent as opposed to how it is currently, which seems more of an internal evaluation of your program, as opposed to useful information others can use to start or improve their own programs. While you have included quotes in the supplementary table, it would be helpful to comment on recurring ideas you have observed in the main body which in the current format, is not clear and seems to be more of individual quotes brought forward by participants. Main emerging themes are not obvious to the reader.**

**Response:** thank you for this important suggestion. We have addressed it by highlighting the themes that have emerged (expectations of residents, coordination, mentorship, and capacity building). We describe the latter three as the ‘pillars’ for a research training for residents, with dedicated subheadings in the results section. We have also added a table (table 1) that describes the elements pertinent to each of these three pillars. Medical educators interested in starting or improving similar programs would hopefully find this information to be useful. We have also replaced figure 2 with another figure that better represents the 3 pillars.

**Reviewer #2**

**Thank you for the opportunity to review " Views of medical residents on a research training program: a qualitative study." This a very interesting topic and is of remarkable importance and clinical relevance. Overall, this a well-structured and concise manuscript. The introduction is short and clear. The methodology is detailed and well explained. The results and discussion are well presented and clarified.**

**Response:** Thank you for the very positive feedback.

**Title: Please check capitalization.**

**Response:** The title has been modified

**Methodology: Page 6, the first paragraph under “overall design and research team – the font size is different than the other paragraphs, please adjust it.**

**Response:** Font size has been adjusted

**Page 7. Sentence 1 under “data collection” section - What is meant by “month to month”.**

**Response:** Apologies for the confusion. We have now specified the exact months when we conducted the data collection)
